# The Family Doctor in the “COVID-19 Era”

**DOI:** 10.3390/healthcare13010032

**Published:** 2024-12-27

**Authors:** Andreea Munteanu, Daniel Florin Lighezan, Maria-Silvia Rosca, Gabriela Otiman, Violeta Ariana Nicoraș, Daciana Nistor, Nilima Rajpal Kundnani, Anca-Raluca Dinu, Ciprian Ilie Rosca

**Affiliations:** 1 Internal Medicine I—Discipline of Internal Medicine IV, Department V, Center of Advanced Research in Cardiology and Hemostasology, “Victor Babeș” University of Medicine and Pharmacy, Eftimie Murgu Sq. No. 2, 300041 Timisoara, Romania; 2 Internal Medicine I—Discipline of Medical Semiology I, Department V, Center of Advanced Research in Cardiology and Hemostasology, “Victor Babeș” University of Medicine and Pharmacy, Eftimie Murgu Sq. No. 2, 300041 Timisoara, Romania; 3 Dr Rosca Civil Medical Society, Teremia Mare Nr 731, 307405 Teremia Mare, Romania; 4 University Clinic of Internal Medicine and Ambulatory Care, Prevention and Cardiovascular Recovery, Department VI—Cardiology, “Victor Babeș” University of Medicine and Pharmacy, 300041 Timisoara, Romaniaknilima@umft.ro (N.R.K.); 5 Department of Doctoral Studies, “Victor Babeș” University of Medicine and Pharmacy, Eftimie Murgu Sq. No. 2, 300041 Timisoara, Romania; 6 Department of Functional Sciences, Physiology, Center of Immuno-Physiology and Biotechnologies (CIFBIOTEH), “Victor Babeș” University of Medicine and Pharmacy, 300041 Timisoara, Romania; 7 Centre for Gene and Cellular Therapies in Cancer, 300723 Timisoara, Romania; 8 Research Centre of Timisoara Institute of Cardiovascular Diseases, “Victor Babeș” University of Medicine and Pharmacy, 300041 Timisoara, Romania; 9 Department XVI, Medical Recovery, “Victor Babeş” University of Medicine and Pharmacy, 300041 Timisoara, Romania; 10 Research Center for Assessment of Human Motion and Functionality and Disability, “Victor Babeș” University of Medicine and Pharmacy, Eftimie Murgu Square, No. 2, 300041 Timisoara, Romania; 11 “Pius Brinzeu” Emergency Clinical County Hospital, Bld Liviu Rebreanu, No. 156, 300723 Timisoara, Romania

**Keywords:** COVID-19, family medicine, family doctor, general practitioner, COVID-19 pandemic

## Abstract

The SARS-CoV-2 virus infection, the most severe pandemic in recent human history, found healthcare systems around the world more or less unprepared. Adjusting to this challenge involved changes in the daily routines of healthcare systems, as well as the patients, once again highlighting the importance of primary care (family physician or general practitioner). In the context of the COVID-19 pandemic, the family doctor in Romania played a crucial role in patient management, rapidly adapting to the changes and challenges imposed by the state of emergency. Their involvement quickly evolved from in-person consultations to online assessments, as they took on responsibilities such as monitoring patients in isolation or quarantine and issuing necessary medical leaves. Moreover, family doctors were directly involved in the COVID-19 vaccination process, facing challenges related to access to scheduling platforms and limited resources of protective equipment. Although they were on the front line of the healthcare response, recognition through incentives or compensations came late and incompletely, and their efforts in combating the pandemic were often overlooked. Designating family doctors’ offices as public utility medical units (regardless of their organisational form) and supporting their activities through increased equipment and medical devices provided by local or central authorities are the keys to fighting for human lives in critical situations. Implementing clear and universal rules regarding the competencies (skills) and duties of family doctors, both in normal life situations and in exceptional circumstances, is of utmost importance. Little is known about the dedicated work and dedication of family physicians to their patients. Few studies have been carried out on the activity of the family doctor and their professional difficulties during the pandemic period. Some studies, on a small number of subjects, tried to evaluate the psychological adaptation of the family doctor to the new epidemiological situation. The aim of this narrative review is to highlight the difficulties to which family doctors had to adapt, comparing the data from the Romanian medical system with those discovered in the medical literature regarding family doctors from all over the world.

## 1. Introduction

The SARS-CoV-2 virus infection, the most severe pandemic in recent human history, found healthcare systems around the world more or less unprepared, both in terms of therapeutic approaches to infectious diseases and in terms of reporting practices that would allow decisions to be made to alleviate deficiencies in the health systems [1]. The humanitarian tragedy that engulfed the entire world will leave deep marks in people’s memory, even if many try to forget it. By the time the WHO declared the end of the pandemic period, almost no family had been untouched by this virus or escaped its consequences. Romania’s healthcare system was severely tested and had to adapt quickly, often on the fly, to this new challenge [2]. The epidemic situation generated by the COVID-19 virus led to the adaptation of the outpatient evaluation system from in-person consultations to online consultations. Family doctors were assigned new responsibilities and duties amidst limited resources (financial, equipment, and especially staff) in an environment where their professional life had previously been restricted and limited by the Romanian National Health Insurance (RNHI) system, including even the prohibition of certain skills and professional rights earned through university training and subsequent residency. For example, family doctors were not allowed to prescribe certain medications on a reimbursed basis by the RNHI without a recommendation from a specialist, such as a cardiologist, internal medicine doctor, pulmonologist, diabetologist, neurologist, or psychiatrist (e.g., medications like clopidogrel, diabetes medication, inhaled bronchodilators, and psychiatric or neurological medications). Unfortunately, the toll of COVID-19 in Romania at the close of the pandemic period showed massive numbers. At the time of the pandemic’s official end, Romania had recorded 68,233 deaths, 3,407,485 cases of COVID-19 infection, and 13,903,394 RT-PCR tests performed [3].

The aim of this review was to highlight the overall situation of family medicine doctors in Romania and worldwide, and their role during such unprecedented situations, against the background of the existence of a small number of articles that evaluate the activity and challenges faced by the family doctors. New guidelines should be built that can help in the implementation of health policies that can effectively regulate desperate epidemiological situations like the one during the COVID-19 pandemic. To carry out this narrative review, the articles published in the period 2020–2024 (up to the time of this review) indexed in PubMed, Google Scholar, and ResearchGate were consulted that responded to the following items to be searched for: “COVID-19 + family doctors/general practitioners + the analyzed country”. The articles taken into analysis were those published in medical specialty magazines or on the official websites of institutions authorized to issue press releases and which were involved in the coordination of medical activity during the COVID-19 pandemic. Non-medical articles were excluded.

Throughout this review, the notion of a family doctor can be assimilated to that of a general practitioner through the prism of the medical activity carried out. With all this, we must not forget the fact that, in many medical systems, family medicine is a medical specialty, and the doctors who are part of these specialists have gone through a training curriculum that has been carried out over several years, such as and by going through training courses in many specialties, as well as having passed a specialty exam that expands their range of competencies and skills compared to general practitioners as simple graduates of medical schools.

## 2. Romania During the COVID-19 Pandemic

The present review represents the experience of a practicing family doctor in Romania during the COVID-19 pandemic.

For the correct framing of events worldwide, the European Centre for Disease Prevention and Control’s (ECDC’s) timeline of the evolution of the COVID-19 pandemic [4] was used with search engines and databases like PubMed, Google Scholar, the website of the Romanian Ministry of Health, and the Internet using the phrase COVID-19 timeline, COVID- 19 evolution cases.

In order to frame the activity of the Romanian family doctor, the websites of the Romanian National Health Insurance Company and the Romanian Ministry of Health were investigated in order to respect the order of events and the legislative changes that regulated his activity during the pandemic.

In order to be able to understand the effort made by the Romanian family doctor, we compared their activity with that of the data reported by other family doctors from around the world based on specific searches using the phrase “family doctors, COVID-19, country” in PubMed, Google Scholar, and ResearchGate. For a comparison with the other countries, we chose the countries whose health systems were deeply affected by the COVID-19 pandemic and also the data from the available literature because, unfortunately, the activity of family doctors during this period is too little reported. From the space limit, we will opt for the most representative countries of each continent, but also for countries whose health system organizations can be superimposed on the Romanian health system with similar attributions and skills of family doctors or general practitioners.

## 3. Evolution of Global Events

The emergence of SARS-CoV-2 infection (Severe Acute Respiratory Syndrome Coronavirus) in China at the end of 2019 seemed initially to affect no one else but China. On 31 December 2019, the Wuhan Municipal Health Commission in Hubei Province, China, reported a cluster of patients (including seven very severe cases) with a new form of pneumonia of unknown origin [4]. Worldwide, people watched, somewhat detached, thinking, “It won’t happen to us”. Even those who took action only simulated coherent measures.

On 17 January 2020, the first information appeared about the transmission risk of the new coronavirus infection, noting a high risk associated with visiting Wuhan’s fish markets, with no clear connection to the source of infection or the mode of transmission [5]. At this time, human-to-human transmission was considered unlikely. Travel to and from China was rated as low risk if it did not involve visits to animal markets or veterinary offices, with the import risk also being considered low [5]. Nosocomial transmission was also deemed low, as no cases were reported among healthcare workers in China treating patients with the new pneumonia type [5]. Based on epidemiological uncertainties at this time, transmission in aircraft was also considered unlikely, and it was recommended that investigations of travelers be limited to those seated on the same row and two rows ahead and behind the suspect case [5].

On 20 January 2020, three more countries joined China in the fight against the new coronavirus, since Thailand, Japan, and South Korea reported cases [6].

On 22 January 2020, with no declared cases, the Romanian government announced its first preventive measures against the new coronavirus, designating infectious disease hospitals from Timișoara, Cluj-Napoca, Constanța, Iași, and Bucharest (with two hospitals) to be responsible for treating patients detected with the new coronavirus [7].

The release of the first official information for health professionals in Romania was marked on 23 January 2020 [8] and constantly updated as the epidemiological data evolved, with the first update on 24 February 2020.

As time passed, old Europe began to tremble. On 24 January 2020, France reported to the WHO European Office the first three patients infected with the new COVID-19 after returning from a Wuhan, China, trip [6]. This was followed by Germany on January 28, 2020, reporting the first cases of pneumonia with the new SARS-CoV-2 virus [6].

On 30 January 2020, the WHO advised its Director-General to declare this situation a public health emergency of international concern [9].

On 10 February 2020, the Romanian National Institute of Public Health issued the first travel alert for trips to and from China, followed by subsequent recommendations for various branches of the Romanian health system [10].

Romania, COVID-19 infection, the healthcare system in general, and family physicians in particular were affected.

On 25 February 2020, the first confirmed COVID-19 case was reported in Romania, an “imported” case in a 71-year-old Italian citizen [11].

On 27 February 2020, the first TELVERDE helpline was set up to inform the general population, although it did not operate non-stop [12].

On 28 February 2020, the first two COVID-19 cases among Romanian citizens were confirmed: a man in Maramureș County and a woman in Timiș County [13].

On 1 March, the first guidelines with recommendations for isolated and quarantined patients, as well as for the rest of the population, were published. Family doctors were designated to issue medical leave for isolation/quarantine based on decisions issued by the County Public Health Departments [14]. This was the beginning of significant challenges for family physicians. Amid the general chaos, it was decided that incapacity-for-work leave certificates would be issued based on a long-awaited form, eventually received after lengthy phone and email exchanges with those responsible for sending the decisions. Transmission methods for these certificates were unpredictable, including options such as emailing the family doctor (a challenging process at the time), sending it to the employer, or personally delivering it through a police officer verifying if the individual was at home. The much-awaited decisions often contained errors and lacked information or signatures. Everyone was agitated and confused, and the authorities responded with minimal action.

Quarantine and self-isolation measures were introduced, accompanied by telephone monitoring by family doctors or the Public Health Department if no family doctor was available [14].

The first elements of preventive public health education for self-isolated individuals were introduced, such as avoiding contact with others, not receiving visitors, handwashing, cleaning and disinfecting surfaces and objects touched, and daily ventilation of rooms [14].

A week after the first confirmed cases of COVID-19, the first general recommendations for healthcare units were issued without specific application based on medical specialties or healthcare unit types [15].

On 8 March 2020, Romania’s National Institute of Public Health (RNIPH or INSPR in Romanian language) published recommendations on the rational use of protective equipment, with a paragraph aimed at helping medical personnel minimize the need for personal protective equipment. A centralizing table (Table 1) offered recommendations for using personal protective equipment based on department, personnel, and type of activity, with guidelines divided between hospital and medical specialty outpatient settings (implicitly including family medicine, but with no specific recommendation) [16]. This material was a partial translation of information provided by the WHO on 27 February 2020 [17].

In this context, a Presidential Decree was issued on 16 March 2020, instituting a State of Emergency for one month, which was subsequently extended for an additional month [18].

New regulations were introduced for medical activities in the national health insurance system starting 16 March 2020 [19]. Notable among these are the treatment of COVID-19 cases regardless of the patient’s insurance status, the discontinuation of the healthcare card requirement for validating medical services, increasing the number of consultations a family doctor can provide to eight per hour of activity (previously number was four patients per hour), and allowing family doctors to prescribe restricted medications from the list approved by Government Decision no. 720/2008, except medications dispensed through closed-circuit pharmacies. This decision aims to reduce patient flow to specialty outpatient clinics, thereby increasing access to family doctors.

Due to the inability to purchase personal protective equipment (masks, disposable gowns, and protective overalls) due to shortages in the supply chain, these items disappeared from suppliers. When those were available, prices were prohibitively high (200 mL of sanitizing alcohol reached RON 50, representing an increase of almost 13 times, and 50 three-layer masks exceeded RON 200, representing an increase of approximately 35 times, with an exchange rate of RON 4.88 to EUR 1). Chlorine was missing from stores, and surface disinfectants and bactericidal solutions were also unavailable from suppliers. These cost increases have not been reflected in the increase in income of family doctors’ offices, with some offices even registering dramatic decreases in income due to the decrease in the addressability of patients for consultation, even for chronic diseases. During that period, the average gross income in Romania was RON 5386, and the net income was RON 3294, according to the National Statistical Institute [20]. Even if it seems that the net income is not directly related to the family doctor, unfortunately, the low value of the salary and the extremely high prices of sanitary alcohol and masks have made it impossible for many Romanians to purchase them according to real needs. So, many of them wore disposable masks for several days in a row. This inadequate preventive behavior contributed to the high rate of spread of the virus.

Additionally, a significant part of the outpatient healthcare system (family physicians and specialty clinics) adapted to technological limitations, implemented “window consultations” instead of telemedicine, which was almost absent, or used online communication platforms for remote consultations but in a limited manner.

On 22 March 2020, the first two deaths were recorded nationwide, at which point there were 433 confirmed cases across the country, 64 of which were declared cured and discharged, with most cases at that time in Timișoara [21].

On 27 March 2020, the National Institute of Public Health of Romania (INSPR) issued a protocol for donning and doffing personal protective equipment in the context of COVID-19 infection [22].

On 27 April 2020, guidelines were issued regarding COVID-19 reporting for family physicians [22].

As cases increased and hospitals reached capacity in treating COVID-19 patients, new regulations stipulated that patients with mild or asymptomatic forms would be treated and monitored by family doctors. If the patient’s condition worsened, they had to call for transport to the hospital by ambulance. The continuously changing treatment protocols made it challenging to synchronize outpatient and hospital treatment practices, and empirical antibiotic therapy, while criticized by many due to the viral origin of COVID-19, was widely adopted by physicians despite the issue of microbial resistance to antibiotics [23]. Antibiotic use for COVID-19 treatment in Romania was below national levels in the United Kingdom. However, Romanian family physicians had approximately 20% higher prescription rates [24].

This last responsibility given to family physicians came without prior training, either in terms of information on COVID-19 infection progression or on conducting remote consultations. Family doctors were required to complete a questionnaire to be administered to patients daily during their 14-day isolation period, in order to track the course of their disease.

To “motivate” family physicians to ensure “remote” medical assistance for isolated patients, it was decided to sign separate contracts with County Health Insurance Houses for payment on a “block” basis for monitoring isolated patients at home. This type of contract was criticized and unsigned by many family doctors.

Another attempt to motivate family doctors was the decision to pay a bonus for the infectious risk and also to the family physicians who were involved in the care of those patients. In August 2020, County Health Insurance Houses sent email notices requesting family doctors to provide proof that they had treated COVID-19 patients or that they or their staff had been infected with SARS-CoV-2 during the state of emergency to qualify for the risk bonus. However, the response period was very short (a few hours) and required conditions that could not always be met (COVID-19 testing was almost nonexistent, and few results reached the family doctors). Those who responded found that the allowance would only go to employees, while most family doctors work independently, often as sole proprietors, not requiring employment contracts. Even under these conditions, payments were delayed (following a second phase of requests) and initially applied only to employees.

The alarming rise in COVID-19 cases prompted the reassignment of specialists from hospital outpatient departments to newly established wards for COVID-19 patients with moderate and severe symptoms, limiting access to specialty clinics for other patients. For chronic patients on treatment regimens including medications previously restricted by the National Health Insurance House for reimbursement through family physicians, the emergency state lifted this restriction, allowing family physicians to monitor patient progress and decide on continuation of treatment. The restrictions previously applied to medications such as clopidogrel, direct oral anticoagulants, allopurinol, sulodexide, inhaled bronchodilators, oral diabetes medications, insulin, levothyroxine, and osteoporosis treatments. The new rules also enabled distance medical consultations through any communication means, requiring documentation of the device or platform used, as well as the exact time of the consultation. Family physicians were again authorized to initiate treatment under the health insurance system without needing referral letters from specialists. These provisions were extended through subsequent decrees by the National Health Insurance House and the Ministry of Health [25].

Restricted access to specialty outpatient departments made managing complex cases difficult for family doctors, particularly newly emerged cases or those with an unpredictable progression, such as complications from COVID-19 infection, post-acute COVID-19 syndrome, or even long-COVID syndrome. Often, decisions had to be based solely on clinical examination and minimal testing due to the limited equipment available in family practice offices. The inability to perform reimbursed investigations within the family practice setting before the COVID-19 pandemic meant that most patients preferred to have these tests done in specialty clinics or hospitals to avoid paying out-of-pocket, leading to limited equipment availability (e.g., ECGs, spirometers, ultrasound, and rapid lab tests) in family practices. Consequently, the quality of medical care suffered.

The fear of hospitalization led some patients to avoid reporting symptoms possibly related to COVID-19, making it difficult to diagnose accurately and delaying treatment according to current protocols while increasing the risk of virus transmission to healthcare personnel working in family physician’s practices.

Family physicians took on new responsibilities after January 2021 by participating in the scheduling of patients for COVID-19 vaccinations using a dedicated platform, which was difficult to navigate, required extensive information entry, and depended on patients having a mobile phone. The platform was practically inaccessible during its initial launch on 16 January 2021. Family doctors, with pre-prepared waiting lists, were forced to access the platform on January 16 and schedule the first patients for phase-two vaccinations [26]. Scheduling continued at the platform’s capacity, and the authorities announced incentive payments for each enrolled patient, later modified to depend on completed vaccinations by patients on the physician’s list. Even under these conditions, payments were fulfilled.

Family physicians continued their dedication to their patients by participating in the activities conducted in the newly established vaccination centers, helping maintain continuous functionality, and, from May 2021, some organized vaccinations within their offices to accommodate patients who could not travel to cities with vaccination centers.

Although the exact number of family physicians who died from COVID-19 infection acquired at work is unknown, some numbers existed for public opinion. It is known that over 30 family doctors died, as announced by Dr. Sandra Alexiu, president of the Bucharest–Ilfov Family Medicine Association, on 2 January 2021 [27].

From 1 April 2022, legislative measures that extended provisions for contracts with County Health Insurance Houses expired, returning patients to increased travel requirements within the healthcare system to address their medical needs [28].

On 28 June 2023, the National Committee for Emergency Situations declared the end of the epidemiological and biological risk status due to COVID-19, marking the closure of structures dedicated to treating or evaluating COVID-19 patients who did not meet the criteria for hospital admission [3].

In Romania, the post-pandemic period brought uncertainty regarding public health policies, amplifying the challenges faced by family doctors. The vaccination campaign suffered from weak and hesitant communication from authorities, leaving family physicians to address patient concerns without adequate support from public health institutions.

Amid the pandemic, family doctors managed a significantly increased patient load, particularly as access to hospitals and specialty clinics was limited for non-urgent cases. They monitored a large number of patients with mild or asymptomatic COVID-19 who did not require hospitalization, providing daily support and remote medical guidance despite lacking sufficient training or resources for this type of care.

Despite these challenges, authorities did not always provide sufficient support for family physicians. Initial shortages of personal protective equipment and bureaucratic hurdles complicated their work, and remuneration and incentive systems were delayed and inefficiently implemented. Furthermore, family doctors had to navigate frequent regulatory changes concerning the treatment and monitoring of COVID-19 patients, complicating workflow.

An ongoing issue was the heavy workload faced by family physicians. Alongside monitoring COVID-19 patients, they continued caring for chronic patients, many of whom had limited access to hospitals and clinics, thus placing extra demands on family doctors, often with inadequate resources to manage both COVID-19 and non-COVID-19 cases concurrently.

Another critical point is the underutilization of telemedicine in Romania during the pandemic. Despite efforts to implement remote consultations, family physicians encountered inadequate digital infrastructure and a lack of clear legislation supporting this type of care. Many patients lacked access to necessary devices or stable internet connections, limiting the effectiveness of these measures.

Additionally, a critical aspect that warrants attention is the lack of psychological support provided to family doctors throughout the pandemic. Many experienced significant emotional stress, facing exhaustion and burnout due to high workloads, uncertainties surrounding pandemic management, and the continual risk of exposure to the virus. Despite these difficulties, no dedicated mental health support programs were available for frontline medical personnel, contributing to general burnout.

## 4. Family Physicians and the COVID-19 Pandemic

The family doctor and his patients are equal partners in a relationship established by a hard-to-destroy bond based on empathy, trust, and hope, which becomes stronger with each visit of the patient to their doctor’s office [29]. This privileged relationship was the basis of the family doctor’s attitude and action throughout the duration of this medical battle. Family doctors, despite their invaluable role in combating COVID-19, encountered significant barriers in their work. The lack of direct involvement in national and regional decision-making processes and poor collaboration with other institutions involved in managing COVID-19 has left more disappointment among family doctors [30].

In many other countries, family doctors also played an essential role in managing the COVID-19 pandemic, although the challenges and responses varied depending on the national context and healthcare system structure.

The most crucial revelation from this pandemic is the insufficient number of family doctors, echoing the World Health Organization’s recommendations to increase the number of family practitioners [31].

### 4.1. Family Doctors in Europe

In the United Kingdom, family doctors (general practitioners or GPs) were rapidly integrated into the pandemic response and tasked with remotely consulting patients, triaging those needing hospital care, and providing palliative care [32,33,34,35]. The National Health Service (NHS) implemented telephone and video consultations to reduce virus transmission risks and manage limited resources, but these methods proved highly dependent upon the patients’ socioeconomic status, marginalizing those with limited financial means [33,36]. Family doctors also played a vital role in the vaccination campaign, showing nearly 97% adherence to complete vaccination themselves, exemplifying their dedication to public health [37], mainly in vulnerable communities [33]. Government interventions aimed at improving the quality of life for GPs were ineffective regarding mental health support, leading to a significant increase in burnout rates [33,38,39]. The unwarranted use of antibiotics among UK GPs was lower than in other countries, although it exceeded that of nations like Romania by approximately 10% at the broader healthcare level [24].

Italy, which saw the most considerable decline in family doctors in Europe over the past 20 years [31], was one of the most affected countries in Europe, and family doctors were on the frontline from the early stages, as hospitals became overwhelmed with severe cases [40]. Family doctors managed mild-to-moderate COVID-19 cases and played a crucial role in home monitoring for isolated patients. Additionally, maximizing hospital facilities led to added responsibilities for family doctors, including continuing cancer screening programs, which proved highly effective [41]. Moreover, contacting high-risk vulnerable individuals to prevent COVID-19-related complications was a successful initiative attributable to GPs [42]. However, the Italian health system faced criticism for poor coordination and difficulty in providing protective equipment, resulting in a high rate of infections and concerning levels of burnout among GPs [43].

In France, family doctors took on similar roles, monitoring and managing patients with mild and moderate COVID-19 infections, including post-infection follow-ups, but their responses revealed a lack of national guidelines and significant practice variability [44,45]. A study evaluating the professional behavior of around 3000 GPs found that responses to COVID-19 took one of several forms: autonomous reorganization (about 65%), inter-specialty reorganization (approximately 16%), reliance on hospitals (5%), and collaboration with COVID-19 outpatient centers (around 15%), highlighting the need for national protocols in crises [46]. Telemedicine was swiftly adopted, and family doctors contributed substantially to prevention and vaccination campaigns [44]. Like other European countries, family doctors in France initially faced difficulties due to a lack of protective equipment and inconsistent communication from authorities. Once these issues were addressed, and particularly after the vaccine rollout, the risk of COVID-19 exposure among family doctors was comparable to that of the general population following the third national infection wave [47]. However, studies indicated that, by the end of the second wave, French GPs experienced more severe mental health impacts, with over 80% reporting psychological distress and more than 70% reporting burnout [48]. Another study found that GPs and patients reported a decline in doctor–patient relationship quality, with greater emotional distance, especially for relationships that were already fragile before the pandemic [49].

In Germany, family doctors were an integral part of the pandemic control efforts, supported by a well-organized system with more substantial resources than in other countries. Nearly 90% of German patients with suspected or confirmed COVID-19 were treated by family doctors [50]. Telemedicine consultations were quickly introduced, and family doctors managed most non-hospitalized COVID-19 cases, with robust governmental support for protective equipment and financial compensation. Despite the support, almost 45% of GPs felt abandoned by those leading the pandemic response [50]. Additionally, 25% of German doctors were explicitly asked to serve in additional roles [50,51]. German patients continued their routine visits for non-COVID health issues, demonstrating a high level of healthcare utilization [52]. The vaccination campaign was efficiently coordinated, with extensive involvement from family doctors in vaccine administration. Self-confidence is one of the most important assets when dealing with unforeseen or challenging situations. A study published in June 2023, based on data collected from seven countries (Austria, Australia, Switzerland, Germany, Hungary, Italy, and Slovenia), showed that female GPs reported feeling less competent and self-assured and also expressed greater fear of infection or transmission to others compared to male GPs [53].

### 4.2. Family Doctors in USA

In the United States of America, family doctors played an essential role in managing the pandemic, but responses varied significantly between states and communities. In rural areas, family doctors were often the first line of defense against the virus, managing patients with limited resources and difficult access to hospitals. Family doctors in many vulnerable communities were vital in promoting vaccination and providing information on preventive measures. However, the pandemic highlighted the inequalities in the American healthcare system, as many family doctors faced financial challenges and a lack of adequate resources.

### 4.3. Family Doctors in Asia

In Asia, family doctors played a critical role in combating the COVID-19 pandemic, although approaches and available resources varied significantly among countries based on healthcare system infrastructure and organization.

In China the initial epicenter of the pandemic, family doctors were involved in the initial triage of patients and in monitoring communities under strict quarantine. The Chinese healthcare system quickly shifted to a centralized model, where patients with severe COVID-19 symptoms were sent to designated hospitals, while mild or suspected cases were managed locally by family doctors and other healthcare staff. Despite a highly efficient resource mobilization effort, family doctors were often overwhelmed, especially in the early stages of the pandemic when information about the virus was limited and protective equipment was in short supply. Although at the beginning of the COVID-19 pandemic, general practitioners (GPs) were not inclined to change their specialty, they were more interested in improving their theoretical and practical training, as well as the services offered to patients [54] However, by mid-2022, there was a drastic shift in intentions as the appeal of the specialty diminished considerably [55]. A study using questionnaires distributed to GPs in Hebei Province in February 2020 demonstrated the importance of improving anti-epidemic protective measures, covering the necessary sleep requirements, and offering resilience-building courses to ensure the mental and psychological well-being of GPs, allowing them to cope with these new challenges [56]. In Hong Kong, the anti-epidemic measures implemented by decision-makers were highly beneficial in limiting human-to-human transmission, and maps pinpointing the location of cases facilitated epidemiological investigations conducted by GPs [30]. Their work system was predominantly private (over 70% of GPs in Hong Kong operate as independent practitioners), which led to limited resources [30]. GPs were involved in testing patients suspected of COVID-19. Those who tested positive were admitted to hospitals, while others remained under GP supervision [30].

In South Korea, family doctors (although limited in number, with 8682 GPs registered in 2020) [57] played a crucial role in testing and monitoring strategies as part of a nationally coordinated effort. Due to South Korea’s well-organized healthcare system and advanced technology, the country rapidly implemented mass testing and contact-tracing mobile applications to monitor virus spread and identify contacts [58]. Family doctors were directly involved in triaging patients and coordinating home treatment for those with mild symptoms. The government provided effective support, including protective equipment and resources, contributing to exemplary pandemic management. The introduction of remote consultations at the end of February 2020 significantly reduced in-person visits, thereby limiting human-to-human transmission [59]. The existence of community (family) pharmacies and the close collaboration between these pharmacies and general practitioners also contributed significantly to limiting virus transmission [60]. Nevertheless, the COVID-19 pandemic highlighted the need to strengthen primary healthcare and increase funding for this sector, along with rethinking and improving the quality of primary healthcare services [57].

In Japan, family doctors played an essential role in managing the pandemic, especially since the Japanese government did not initially implement clear health policies or standards, leaving the population to make decisions based on personal knowledge of how to deal with the infection [61]. General medical practitioners were able to act as primary healthcare providers in local communities, monitoring patients who did not require hospitalization. Given their organizational model, which relies on strong knowledge of residents within their designated areas, GPs were effectively able to respond to patient needs [61]. The Japanese healthcare system’s structure, based on cooperation between local clinics and large hospitals, enabled a structured response, often driven by GPs’ initiatives to create new connections among various epidemic actors. The highly practical approach they took in responding to immediate medical needs at each stage of the COVID-19 pandemic demonstrates the immense adaptability of primary care physicians [61,62]. The use of antiviral drugs by GPs was not particularly significant [63]. Although initially hesitant to adopt mass testing, family doctors took a significant role in educating patients, managing mild cases, and implementing preventive measures. However, as the pandemic continued with successive waves, the pressure on medical resources and personnel grew significantly, increasing outpatient consultations and directing more patients to pharmacies for over-the-counter medications and treatments [64].

In India, where the pandemic hit especially hard during the second wave, family doctors played a critical role in managing patients at the community level, particularly in rural and impoverished areas with limited access to hospitals and advanced medical resources [65]. In the initial pandemic phase, in-person medical consultations decreased by approximately 70% [66]. India’s healthcare system adapted to telemedicine “on the go”, since this type of medical care was neither well-regulated nor widely used in the country. Similar limitations were encountered with telephone consultations [67,68]. Family doctors were responsible for early diagnosis, symptom monitoring, and home-based treatment management, often without adequate resources, such as protective equipment and oxygen supplies [69]. In April 2020, to support family doctors, The Academy of Family Physicians of India developed a position paper guiding family physicians in the fight against this pandemic [70]. Despite the heroic efforts of family doctors, the Indian healthcare system was overwhelmed, and logistical and financial support shortages led to a deep crisis in pandemic management. India’s healthcare system’s experience with the COVID-19 pandemic has drawn attention to the need to strengthen primary healthcare by increasing the number of doctors and training them in the specific aspects of family medicine [71].

In Singapore, an exceptionally well-organized healthcare system enabled an efficient response to COVID-19, drawing on the lessons learned from the H1N1 influenza pandemic [72]. The number of consultations for acute and chronic diseases dropped significantly in the early stages of the COVID-19 pandemic, as patients hesitated to use GP services [73]. Family doctors played a major role in prevention and patient management, and online consultations and telemedicine were widely adopted. With substantial government support, including a steady supply of protective equipment and an efficient vaccination campaign, family doctors managed to keep the virus’ spread under control and provide quality care to patients [72]. However, the perception of their profession changed post-pandemic, with approximately 15% of GPs considering leaving their practice, about 13% wanting a professional break, and over 50% planning to reduce their working hours. This shift reflects the dissatisfaction stemming from their perceived role in society and within the medical community, where general and family medicine are not viewed as medical specialties [74].

In the Philippines, the primary care segment of the public health system is funded by local government resources [75]. Filipino family doctors faced significant challenges, particularly in rural and island communities with limited access to healthcare. They had to manage COVID-19 cases with limited resources in an underfunded healthcare system, facing shortages of equipment and often inadequate government support, while state funding for private GPs was absent, leaving patients to pay out-of-pocket for private services [75,76]. Despite these challenges, Filipino family doctors continued providing essential healthcare, serving as a crucial support in local communities. Telemedicine in primary care was supported by government collaboration with the Academy of Family Physicians, allowing GPs to offer free consultations through this system [75]. They were also involved in epidemiological screening and quarantine activities at the country’s borders alongside other public health professionals [75]. The adoption of an online telepharmacy played a complementary role in supporting primary healthcare providers by clarifying prescribed treatments and recommended preventive measures [77].

### 4.4. Family Doctors in Africa and Arab Countries

In Africa and the Arab countries, family doctors played an essential role in managing the COVID-19 pandemic, but the challenges were amplified by structural weaknesses in the healthcare systems, limited resources, and their uneven distribution.

In South Africa, one of the hardest-hit African countries, family doctors were crucial in coordinating community-level care, though they initially had access to inaccurate information, leading to a negative perception and understanding of the pandemic [78]. Telemedicine became an essential tool during the pandemic and retained its usefulness after the pandemic ended [79]. Alongside managing COVID-19 cases, family doctors continued treating prevalent chronic illnesses like HIV/AIDS and tuberculosis, complicating the pandemic response. They were also involved in public education campaigns, vaccination, and monitoring of isolated patients at home. However, shortages of personal protective equipment and adequate medical resources placed enormous pressure on the healthcare system [80]. When assessing the healthcare system’s needs after the pandemic, it became clear that the clinical and educational requirements of family doctors were not uniform, as their skills varied significantly across specialties [81]. Some doctors expressed fear at the idea of providing potentially incorrect information to those seeking specialized advice to help navigate this challenging epidemiological period [82].

In other African countries, such as Nigeria and Kenya, family doctors faced considerable challenges due to a lack of medical infrastructure, particularly in rural areas. While responsible for managing COVID-19 cases and educating the community, family doctors faced a shortage of personal protective resources, testing, and treatment options, making it challenging to monitor and manage patients effectively [83,84]. Although Nigerian authorities responded quickly, their support was insufficient to address the additional epidemiological demands, as Nigeria’s healthcare system was already strained by pre-existing issues [85]. Additionally, in many African countries, vaccine access was problematic, with family doctors leading vaccination efforts whenever resources became available. The significant reallocation of healthcare resources to fight COVID-19 meant that infectious tropical diseases continued at alarmingly high rates despite the new public health threat [83]. The burnout syndrome incidence rose alarmingly among medical staff, and family doctors were no exception, reflecting the immense effort required [86]. Telemedicine was hardly accessible to GPs for reasons including a lack of legal regulations and insufficient financial resources for access [87]. In Kenya, GPs, even underfunded, were involved in all four strategic axes of the fight against COVID-19, namely public awareness, home-based care, population screening, and treatment for cases not requiring hospital referral for advanced care [88]. Faced with challenges due to limited information on COVID-19 and a shortage of protective equipment, which led some GPs (who are GPs without graduating from a program in GP specialization) to refuse to evaluate patients with flu-like symptoms out of concern for their own safety [88,89]. As in many other countries, the immense psychological stress on healthcare workers went largely unsupported by authorities [90]. The growth of virtual medical consultations was slow. By February 2021, only 20 medical facilities had been authorized to conduct such consultations [91].

In Egypt, GPs (doctors without graduating from a program in GP specialization) and family doctors played a critical role in managing COVID-19 cases, especially in the initial phases of the pandemic when hospitals were overwhelmed [92]. They provided community-level care, monitoring patients with mild or moderate symptoms at home, thereby reducing the strain on hospitals. Additionally, they contributed to national vaccination campaigns despite logistical challenges and the phased vaccine rollout based on risk prioritization [92]. The pandemic underscored the need for better training in infection control measures [93]. The emotional and physical stress endured by Egyptian doctors during this period was enormous [94,95]. Telemedicine was utilized, but its potential was not fully realized, indicating a need to build trust in virtual consultations [96].

In Morocco, family doctors were integrated into the national COVID-19 response system, where they played a vital role in testing, triaging patients, and administering vaccines. Morocco’s healthcare system managed the crisis relatively well, largely due to an organized vaccination campaign that demonstrated a reduced infection risk among healthcare workers following vaccination [97]. Initially, telemedicine was informal, but it was soon formalized to ensure the continuity of healthcare [98]. Doctors fighting the COVID-19 pandemic have seen a decrease in their quality of life, although this decrease is more noticeable among female doctors [99]. Due to a significant amount of work, numerous GPs experienced severe symptoms of physical and mental exhaustion [100].

In Tunisia, family doctors were actively engaged in managing the pandemic, using remote consultations and monitoring patients in home isolation. However, they were sidelined by the decisions of the national COVID-19 response committee [101]. They also played a role in the national vaccination campaign, but resource limitations in protective equipment and healthcare infrastructure posed challenges. Like other countries, the overwhelming workload during the severe COVID-19 waves led to significant physical and psychological strain among family doctors. The pandemic highlighted, through physical and psychological stress of the GPs, the need for a re-evaluation of healthcare policies and the necessity of providing psychological support to healthcare workers facing high-stress situations [102].

In Saudi Arabia, the government implemented swift and stringent measures to curb the virus’ spread, and family doctors were crucial in community monitoring and patient evaluations for mild symptoms. Nevertheless, some GPs encountered confusion or a lack of information about COVID-19 due to frequently changing recommendations or multiple sources of guidance [103,104]. The Saudi healthcare system, benefiting from significant resources, saw an organized and effective vaccination campaign, in which family doctors played an important role. Chronic disease consultations decreased, and the duration of consultations was shortened Additionally, technology and telemedicine were expanded to minimize direct contact, although this sometimes delayed accurate diagnoses and prescription issuance [103,105]. The emotional impact of the pandemic on healthcare workers was significant, with many experiencing anxiety, stress, and even obsessive–compulsive symptoms [106,107].

In the United Arab Emirates (UAE), family doctors were integral to a well-coordinated strategy for mass testing, community monitoring, continuity of care, and vaccination [108]. Within a well-funded healthcare system, family doctors received sufficient resources and logistical support to effectively respond to the crisis [109]. Medical professionals involved in treating COVID-19 patients, including family doctors, were tested every two weeks [108]. Like in other countries, face-to-face consultations for various medical conditions decreased across healthcare sectors [110]. Telemedicine and digital technology were widely used to maintain primary care, while the vaccination campaign was implemented swiftly and with impressive outcomes. The importance of vaccination was firmly embedded in the memory of healthcare professionals, with family doctors being among the most proactive supporters of vaccination, even after the pandemic [111].

The main lesson learned from the end of the COVID-19 pandemic is that, for a nation to survive, it requires a well-funded healthcare system with highly trained human resources that have access to information, cutting-edge medical equipment, and technology. This system’s leadership must have a comprehensive vision and include family doctors at all decision-making levels, as they are the first point of contact for people in distress, regardless of the cause or the patient’s socioeconomic status [85].

## 5. Conclusions

Little is known about the involvement of family doctors in the fight against COVID-19, with their engagement and dedication receiving limited media coverage or analysis in studies generated by the pandemic. Even less is understood about their perception of the struggle they faced, their understanding of their roles, and the aspects that should be improved to ensure that future challenges of similar magnitude result in far fewer casualties.

Family doctors from various countries played a fundamental role in managing the COVID-19 pandemic, ensuring the continuity of primary care, patient triage, and support for vaccination campaigns. However, the level of preparedness, government support, and resource access varied considerably, impacting their ability to respond effectively to the challenges posed by the pandemic.

The family doctor represents the “cornerstone” of any healthcare system. The role of family doctors underwent profound changes during and after the COVID-19 pandemic. Their high adaptability to an ever-evolving situation, coupled with their ability to step in for other medical specialties to maintain or restore their patients’ health, was exemplary.

Thus, the pandemic underscored the crucial importance of the family doctor within Romania’s healthcare system, not only as the first point of contact for patients but also as an essential resource in managing public health crises. Despite the difficulties encountered, their role was vital for maintaining the functionality of the healthcare system during periods of extreme stress.

The state of health systems pre-pandemic can be summarized as follows. The family doctor, even if he is the first-contact professional for the patient from the point of view of the patient’s relationship with the health system, is often avoided, preferring evaluation by the other specialists in the medical system. The endowment of medical offices is left exclusively to family doctors from financial sources resulting from their direct activity, and the acquisition of medical or paramedical materials and supplies also falls under the care of the family doctor (both administratively and financially). The payment of the medical and non-medical staff of the medical office is also made from the income of the family medicine office.

There is a lack of financial involvement of state or local communities in the direct financing (unrelated to medical care) of family medicine offices. These can limit the professional skills and the possibility of recommending medical investigations or treatments, limiting the number of consultations that the doctor can offer in a day.

Furthermore, there is a permanent and unpredictable change in legislation that regulates the activity of the family doctor. Another demerit is the unjustified change in the various standardized forms used in the medical system. There is a direct and real non-involvement of family doctors in the decision-making that concerns their professional activity (the professional societies of family doctors have an advisory role, being deprived of negotiation power).

The lack of population awareness programs on infectious risk can be considered an additional hurdle. Similarly, there is a lack of coherent and coercive programs and measures that can be applied to situations where adults expose themselves or their children to infectious risks by refusing vaccinations, which are difficult to handle. There are a lack of programs regarding awareness of the fact that the abuse of antibiotics leads to the increase of bacterial resistance.

New directions should be addressed to improve the passage through periods of epidemiological instability. Respecting the professional competencies of family doctors and developing continuing medical education programs to further expand their competencies and skills would be beneficial. Similarly, the creation of medical systems that respect the principle of first-contact medical assistance and that no longer encourage a continuous assessment of patients in specialized medical services could help. The development of health policies that take into account the suggestions of family doctors and the particularities of family medicine would be beneficial. Increasing the number of family doctors, especially in isolated or economically underdeveloped localities, by developing attractive financial programs that allow for the equipment and operation of newly established offices, with an increase in the financial benefits for family doctors who will choose to carry out their activity in those communities, can further help. Detailed and staged action plans for special situations (be they epidemiological, natural calamities, armed conflicts, or other special situations) in which family doctors are also involved at the decision-making level should be established, as well as the establishment of color and special mobility circuits in special situations for medical assistance, along with the allocation of funds to enable the equipping and maintenance of the devices in the endowment of family doctors’ offices.

Create stocks and inventories of medicines, sanitary materials, and equipment that can be distributed immediately in special medical situations. The “remote” diagnostic skills that are compromised by remote settings can be improved by increasing the use of online or remote consultations that reduce the inability of patients with infectious-contagious pathology to obtain advice to help combat the symptoms associated with the infection, as well as for the monitoring of patients with chronic diseases.

Having a setup that is environmentally friendly, allows for the easy circulation of information digitally, and avoids the use of physical copies of paper-type forms (prescriptions, referral tickets, and the patient’s medical record) can prove to be more efficient and time-saving. Furthermore, establish community pharmacies capable of ensuring the distribution of medicines in a “non-stop” regime in all territorial administrative units with the possibility of establishing a larger number of pharmacies for cities with many inhabitants distributed uniformly in all large neighborhoods.

Considering all of the above-mentioned aspects, family doctors, along with other specialists involved in combating this pandemic, truly deserve the ungranted title of “frontline fighter”!

## Figures and Tables

**Table 1:** Personal protective equipment according to [16].

PPE Type to Use	Close Contact (Less than 1 m Away) with a Possible Patient		Contact with a Confirmed COVID-19 Case
	Without GAP	GAP	
Hands hygiene	Yes	Yes	Yes
Gloves	Yes	Yes	Yes
Single-use water-proof apron	Yes	No	No
Single-use protective coat with long sleeves	No	Yes	Yes
3 layers surgical facial mask	Yes	No	Yes
FFP3 mask	No	Yes	No
Eyes protection	Self-risk evaluation	Yes	Yes

PPE—personal protection equipment, GAP—generating aerosols procedures.
